# Efficacy of functional electrical stimulation at different frequencies for post-stroke foot drop: a retrospective cohort study

**DOI:** 10.3389/fneur.2026.1766337

**Published:** 2026-03-10

**Authors:** Bin Zhang, Chen Fang, Youqing Su

**Affiliations:** Department of Rehabilitation, Hangzhou Ninth Hospital, Hangzhou, China

**Keywords:** foot drop, frequency, functional electrical stimulation, rehabilitation, stroke

## Abstract

**Objective:**

To compare the efficacy and safety of low, medium, and high-frequency functional electrical stimulation (FES) in post-stroke foot drop (FD), aiming to identify optimal frequency parameters for clinical practice.

**Methods:**

In this retrospective cohort study, 90 patients with post-stroke FD admitted between January 2021–December 2023 were grouped based on received FES frequency: low-frequency (20–30 Hz, *n* = 30), medium-frequency (31–40 Hz, *n* = 30), and high-frequency (41–50 Hz, *n* = 30). All patients received conventional rehabilitation combined with FES. The primary efficacy outcome was the improvement in 10-meter maximum walking speed (10MWS). Secondary outcomes included Fugl-Meyer Assessment for Lower Extremity (FMA-LE) scores and Functional Ambulation Category (FAC), and ankle dorsiflexor muscle strength.

**Results:**

Baseline characteristics were comparable (all *p* > 0.05). The medium-frequency group showed significantly greater improvement in 10MWS (0.246 ± 0.095 m/s) versus low (0.154 ± 0.063 m/s) and high-frequency groups (0.145 ± 0.050 m/s) (*p* < 0.001). FMA-LE improvement was also superior in the medium-frequency group (8.60 ± 1.99 points; *p* < 0.001). Ankle dorsi-flexor strength improvement was significantly greater in the medium-frequency group (1.93 ± 0.25 grade) compared to both low (0.97 ± 0.18 grade) and high-frequency groups (1.03 ± 0.18 grade) (*p* < 0.001), with a favorable trend also observed in FAC. Adverse event incidence was low (3.3%) and similar across groups (*p* = 0.355). Subgroup analysis indicated consistent medium-frequency efficacy across stroke types.

**Conclusion:**

In this retrospective comparative study, medium-frequency (31–40 Hz) FES was associated with optimal efficacy and safety outcomes for post-stroke FD, showing significantly greater improvement in walking speed and lower limb function without added risk. These findings suggest it as a promising parameter for clinical evaluation, though verification by prospective trials is warranted.

## 1 Introduction

Stroke remains one of the leading global causes of disability and mortality (1, 2). Epidemiological data indicate a significant increase in the overall burden of stroke from 1990 to 2021, reflected by a 70.0% rise in new cases, a 44.0% increase in deaths, an 86.0% growth in prevalence, and a corresponding 32% increase in disability-adjusted life years (3). Stroke-induced impairments extensively affect multiple dimensions, including physical function, occupational participation, communication ability, and social integration (4), with motor dysfunction being particularly prominent; approximately 80% of survivors experience varying degrees of limb motor impairment (5). Foot drop (FD), a common manifestation of motor dysfunction, is characterized primarily by weakness in ankle dorsiflexion and eversion. This leads to diminished mobility, impaired balance, and reduced walking efficiency, subsequently increasing the risk of falls and significantly constraining patients’ quality of daily life and level of social participation (6).

Electrical stimulation is among the effective treatments for alleviating walking dysfunction after stroke. In particular, functional electrical stimulation (FES) has become a standard neurorehabilitation intervention for post-stroke FD (7, 8). This is supported by high-level evidence, such as a systematic review and meta-analysis of randomized controlled trials which concluded that FES significantly improves walking speed in ambulatory stroke survivors (9). This technique involves stimulating the common peroneal nerve via surface electrodes during the swing phase of gait to elicit ankle dorsiflexion, thereby effectively correcting gait abnormalities (10). The underlying mechanisms include FES’s ability not only to stimulate voluntary muscle activity, directly ameliorating FD and reducing spasticity, but also to promote long-term functional reorganization of the sensorimotor cortex (11, 12).

However, despite the established efficacy of FES, optimizing its treatment parameters remains a key challenge in current rehabilitation medicine research. Stimulation frequency, as a core FES parameter, directly influences muscle contraction characteristics, the degree of neuromuscular fatigue, and the ultimate functional output. Although the commonly used clinical frequency range is broad (20–50 Hz), aiming to elicit functional contraction of target muscles by activating motor nerve fibers (11), a consensus on the optimal frequency is lacking and significant controversy persists. Some studies support the use of lower frequencies to achieve smoother muscle contractions (13), while other evidence suggests that higher frequencies offer advantages in improving muscle strength and motor activation characteristics (14). This uncertainty in clinical practice stems primarily from a lack of high-quality studies directly and systematically comparing different frequencies. Indeed, recent reviews have explicitly highlighted the need for further comparisons of the effects of neuromuscular electrical stimulation with different stimulation frequencies (11).

Based on the premise that higher frequencies might generate stronger muscle contractions, we initially hypothesized that high-frequency FES would yield superior functional outcomes compared to low-frequency stimulation. Therefore, this clinical efficacy study aims, via a retrospective cohort design, to systematically compare the functional outcomes of FES at three commonly used frequency ranges (low: 20–30 Hz, medium: 31–40 Hz, high: 41–50 Hz) on walking function, lower limb motor function, and safety in patients with post-stroke FD. The primary goal is to inform optimal parameter selection for clinical practice, rather than to elucidate underlying neurophysiological mechanisms.

## 2 Materials and methods

### 2.1 Study design

This study adopted a retrospective cohort design. It was approved by the Ethics Committee of Hangzhou Ninth People’s Hospital (currently Hangzhou Ninth Hospital) (Approval No.: 2024–051), and informed consent was waived due to the study’s retrospective nature.

### 2.2 Patient selection

We consecutively retrieved medical records of patients with post-stroke FD hospitalized in the Department of Rehabilitation at our hospital between January 1, 2021, and December 31, 2023. Inclusion criteria were: 1) diagnosis of stroke confirmed by imaging; 2) first-ever stroke, with disease in the recovery phase (3 months to 1 year); 3) clear clinical presentation of FD (ankle dorsiflexion muscle strength ≤ grade 3); 4) age between 18 and 80 years; 5) consecutive FES treatment for no less than 4 weeks with complete medical records. Exclusion criteria included: 1) severe cardiac, hepatic, or renal insufficiency; 2) severe contractures or deformities in the lower limbs; 3) presence of internal electronic implants such as cardiac pacemakers; 4) skin breakdown, infection, or severe sensory loss in the affected lower limb; 5) severe cognitive or speech impairment precluding cooperation with assessments; 6) missing clinical data.

### 2.3 Intervention and grouping

By systematically reviewing patients’ rehabilitation treatment records, they were grouped according to the actual FES treatment frequency they received. It is crucial to note that the assignment to a specific frequency was a clinical decision made by the treating therapist during the original course of care, based on factors such as departmental protocol, initial patient response, and clinical judgment. This was not a randomized or research-driven allocation. Therefore, this study represents a retrospective analysis of existing clinical practice patterns. Group definitions were based on a synthesis of the commonly used clinical range and frequency demarcations in existing literature. The overall 20–50 Hz range covers the effective bandwidth commonly used in clinical practice and systematic reviews (11). Within this range, we referred to typical frequency classifications in stroke research. For instance, Doucet et al. (14) compared the efficacy of 20 Hz versus 40 Hz as representative low and high frequencies, respectively, aligning with the rationale of classifying ≤ 30 Hz as low frequency and ≥ 40 Hz as high frequency in this study. Accordingly, this study established a low-frequency group (20–30 Hz, *n* = 30), a medium-frequency group (31–40 Hz, *n* = 30), and a high-frequency group (41–50 Hz, *n* = 30). The initial screening identified 112 eligible patients, with a natural uneven distribution across the three frequency bands. To enable a balanced comparative analysis and mitigate the confounding effect of unequal sample sizes, we constructed the final cohorts as follows: we first included all consecutive eligible patients from the smallest frequency band. Then, for the band(s) with more patients, we randomly selected an equivalent number from their consecutive list to match the size of the smallest group. This methodological step explains the equal group sizes (*n* = 30) for analysis. All FES treatments were delivered using the same device model (XFT-2001E G4, Xiangyu Medical), ensuring consistent stimulation characteristics.

#### 2.3.1 Aim and rationale

The FES intervention in this study was applied with a dual therapeutic purpose: (1) as a functional orthosis to immediately correct foot drop by eliciting ankle dorsiflexion during the swing phase of gait, thereby directly improving walking safety and efficiency; and (2) as a therapeutic exercise modality aimed at providing repetitive, task-specific motor activation to the impaired dorsiflexor muscles. This combined approach targets both immediate functional improvement and potential long-term neuromuscular recovery. Stimulation target: The primary goal of stimulation was to elicit a functional ankle dorsiflexion and eversion movement to correct foot drop. This was achieved by targeting the common peroneal nerve, which innervates the tibialis anterior and the peroneal muscles (e.g., extensor digitorum longus, peroneus longus), thereby producing a synergistic dorsiflexion and eversion response.

#### 2.3.2 Device and stimulation configuration

The FES intervention was delivered using the XFT-2001E G4 Foot Drop Rehabilitation System (Xiangyu Medical). All treatments were delivered using devices of this identical model. While multiple units were employed in the clinical department, the standardized hardware, software, and clinical protocol ensured consistent stimulation characteristics across all patients. This device utilizes a micro-electromechanical systems (MEMS) sensor and a proprietary artificial intelligence algorithm to continuously monitor gait phase. Stimulation was automatically triggered by the sensor, synchronized with the swing phase during walking to elicit ankle dorsiflexion and eversion.

Electrode configuration: The device features an integrated cuff with patented sequenced electrodes (medical-grade stainless steel) designed based on anatomy to accurately cover the peroneal nerve branches, eliminating the need for separate adhesive electrodes. The same cuff was assigned to each patient for the duration of their treatment. It was cleaned with disinfectant after each session. Conductive gel was applied to the electrode surface prior to each use to ensure good skin contact and low impedance.

#### 2.3.3 Stimulation parameters and adjustment protocol

Stimulation parameters: Apart from the frequency grouping under investigation (low: 20–30 Hz; medium: 31–40 Hz; high: 41–50 Hz), other foundational parameters were set as follows: the output waveform was a biphasic square pulse. Pulse width was individually set by the therapist, typically within a range of 220–280 μs (mean ± SD per group shown in Table 1), to optimize patient comfort and motor response. The key therapeutic parameter, stimulation intensity (current amplitude, in milliamperes), was determined and managed following a standardized clinical procedure: (1) Initial Intensity Determination: At the beginning of the treatment course, the intensity was individually titrated for each patient. It was set to the lowest level that elicited a clear, functional ankle dorsiflexion (lifting the foot to at least neutral position) during the swing phase, while ensuring the stimulation remained comfortable and did not cause pain or excessive fatigue (2). Intensity Adjustment Protocol: Throughout the 4-week intervention, the stimulation intensity was dynamically adjusted by the treating therapist. Adjustments were made at the start of a session if: The previous session’s intensity no longer produced an adequate motor response (indicating potential adaptation or improvement). The patient reported discomfort. The therapeutic goal was to challenge the muscle further as performance improved. The adjustment aimed to maintain the “therapeutic window”—a strength sufficient for functional foot clearance without causing discomfort. This dynamic titration ensured that the stimulation remained effective and tailored to the patient’s evolving condition. Typical Range: Based on this protocol, the effective stimulation intensity for the cohort typically ranged between 15–35 mA.

**Table 1:** Comparison of baseline characteristics among the three groups.

Characteristic	Low-frequency	Medium-frequency	High-frequency	Statistic (*p* value)
Age, years	67.53 ± 12.15	61.20 ± 12.46	64.73 ± 12.00	*F* = 2.029 (*p* = 0.138)
Male sex	14 (46.7%)	15 (50.0%)	15 (50.0%)	*χ^2^* = 0.089 (*p* = 0.957)
BMI, kg/m^2^	24.73 ± 3.50	24.77 ± 3.04	24.36 ± 4.54	*F* = 0.112 (*p* = 0.894)
No. of comorbidities	1.33 ± 1.09	1.27 ± 1.05	1.17 ± 1.05	*F* = 0.186 (*p* = 0.831)
Ischemic stroke	21 (70.0%)	24 (80.0%)	23 (76.7%)	*χ^2^* = 0.842 (*p* = 0.656)
Left affected side	18 (60.0%)	11 (36.7%)	11 (36.7%)	*χ^2^* = 4.410 (*p* = 0.110)
Disease duration, days	180.90 ± 106.06	191.60 ± 108.68	207.07 ± 107.60	*F* = 0.450 (*p* = 0.639)
10MWS, m/s	0.44 ± 0.21	0.42 ± 0.20	0.41 ± 0.20	*F* = 0.126 (*p* = 0.882)
FMA-LE score	12.93 ± 5.21	15.90 ± 4.94	15.53 ± 5.83	*F* = 2.753 (*p* = 0.069)
FAC level	1.43 ± 1.10	1.47 ± 0.82	1.33 ± 0.96	*F* = 0.154 (*p* = 0.857)
Ankle DF strength	1.93 ± 1.23	2.00 ± 0.98	1.90 ± 1.35	*F* = 0.054 (*p* = 0.947)
BBS score	25.07 ± 9.72	22.80 ± 9.75	21.80 ± 8.24	*F* = 0.979 (*p* = 0.380)
Treatment duration, weeks	5.00 ± 1.02	5.00 ± 1.02	4.87 ± 1.01	*F* = 0.173 (*p* = 0.842)
Sessions/week	4.90 ± 0.85	4.70 ± 0.92	4.90 ± 0.85	*F* = 0.530 (*p* = 0.591)
Pulse width, μs	253.03 ± 32.67	244.43 ± 28.90	251.20 ± 27.94	*F* = 0.688 (*p* = 0.505)

Data are mean ± SD or *n* (%). BMI, body mass index; 10MWS, 10-meter walking speed; FMA-LE, Fugl-Meyer Assessment for Lower Extremity; FAC, Functional Ambulation Category; BBS, Berg Balance Scale; DF, dorsiflexion. Between-group comparisons used one-way ANOVA (*F*) for continuous variables and chi-square (*χ*^2^) for categorical variables; *p* values are in parentheses.

#### 2.3.4 Treatment regimen

Treatment regimen: All patients received FES therapy using the device in Gait Mode alongside conventional rehabilitation. During each session, patients engaged in functional walking training under therapist supervision. Each session lasted approximately 30 min, administered 5 times per week for a consecutive 4 weeks. The treatment followed a recommended acclimatization schedule, gradually increasing daily usage time.

All patients received standardized conventional rehabilitation therapy in addition to their respective FES regimen.

### 2.4 Outcome measures

#### 2.4.1 Primary efficacy outcome

The primary outcome measure was the 10-meter maximum walking speed (10MWS). This was assessed by measuring the time required for the patient to walk 10 meters safely at their fastest possible speed (including acceleration and deceleration distances), calculated in meters per second (m/s). It is an objective and sensitive indicator for evaluating improvement in walking capacity in stroke patients (15).

#### 2.4.2 Secondary efficacy outcomes

Secondary outcomes included the following three aspects:

Lower limb motor function: Assessed using the Fugl-Meyer Assessment for Lower Extremity (FMA-LE), which comprehensively evaluates reflexes, synergistic movements, isolated movements, and coordination of the lower limb (16).

Functional walking capacity: Assessed using the Functional Ambulation Category (FAC), which rates the level of assistance required and walking independence in daily life (17, 18).

Ankle dorsiflexor muscle strength: Assessed manually and graded according to the Medical Research Council scale (ranging from 0, no contraction, to 5, normal strength), focusing on the strength of the ankle dorsiflexors.

#### 2.4.3 Safety outcomes

Throughout the treatment period, all potential adverse events related to FES treatment were closely monitored and recorded, such as skin rash, pain, discomfort, or muscle fatigue at the electrode sites, to evaluate the safety and tolerability of the different stimulation frequency protocols.

### 2.5 Statistical analysis

All data analyses were performed using SPSS software (version 26.0). Normality of continuous variables was tested using the Shapiro–Wilk test. Normally distributed continuous data (e.g., age, scores, walking speed) were presented as mean ± standard deviation (mean ± SD). Intra-group pre-post comparisons were performed using paired *t*-tests. Inter-group comparisons (including baseline and improvement values) were performed using one-way analysis of variance (ANOVA). If ANOVA results were significant, the least significant difference (LSD) method was used for post-hoc pairwise comparisons. For non-normally distributed continuous data or ordinal data (e.g., FAC), intra-group comparisons used the Wilcoxon signed-rank test, and inter-group comparisons used the Kruskal-Wallis H test, with post-hoc pairwise comparisons performed using the Mann–Whitney U test. Categorical data were presented as counts (percentages), and inter-group comparisons were performed using the chi-square test (*χ^2^*) test or Fisher’s exact test (when expected counts were < 5). The significance level for all statistical tests was set at *p* < 0.05.

Specific tests for each outcome measure were applied as follows:

10-meter maximum walking speed (10MWS) and Fugl-Meyer Assessment for Lower Extremity (FMA-LE) scores: These continuous, normally distributed data were analyzed using paired t-tests for within-group (pre *vs.* post-treatment) comparisons and one-way analysis of variance (ANOVA) followed by Least Significant Difference (LSD) post-hoc tests for between-group comparisons of baseline values and improvement scores.

Functional Ambulation Category (FAC): This ordinal scale was analyzed using the Wilcoxon signed-rank test for within-group comparisons and the Kruskal-Wallis H test followed by Mann–Whitney U tests for *post-hoc* pairwise between-group comparisons.

Baseline characteristics (e.g., age, BMI, disease duration): Normally distributed continuous variables were compared using one-way ANOVA; non-normally distributed variables were compared using the Kruskal-Wallis test; categorical variables (e.g., sex, stroke type) were compared using the chi-square test or Fisher’s exact test.

## 3 Results

### 3.1 Comparison of baseline patient characteristics

A total of 90 patients with post-stroke FD were enrolled and divided into low, medium-, and high-frequency groups based on FES stimulation frequency, with 30 patients in each group. As shown in Table 1, there were no statistically significant differences among the three groups in general characteristics such as age, sex, body mass index (BMI), number of comorbidities, stroke type, affected side, or disease duration (all *p* > 0.05). Before treatment, baseline levels of key efficacy indicators, including 10MWS, FMA-LE score, FAC, ankle dorsiflexion muscle strength, and Berg Balance Scale (BBS) score, were also comparable across groups (all *p* > 0.05). Furthermore, there were no significant differences in treatment parameters such as treatment duration and treatment sessions per week among the three groups (all *p* > 0.05). As part of the individualized FES parameter optimization (detailed in Section 2.3.1), the pulse width was titrated within a defined range. The mean pulse width used did not differ significantly across groups (*p* = 0.505, Table 1), indicating that this parameter was balanced and not a source of systematic bias in the comparison.

### 3.2 Comparison of efficacy outcomes

In reporting the following analyses, we adopt a tiered approach to interpretation: findings supported by statistically significant omnibus tests (*p* < 0.05) are considered primary evidence, while those derived from exploratory post-hoc analyses following non-significant overall tests or from underpowered subgroups are clearly labeled as such and interpreted with appropriate caution.

#### 3.2.1 Primary efficacy outcome

The 10MWS was comprehensively evaluated pre and post-treatment in all three groups. As shown in Table 2, after 4 weeks of combined FES and conventional rehabilitation, walking speed improved significantly in all patients (paired *t*-test, all *p* < 0.001). Preliminary analysis revealed a significant inter-group difference in the magnitude of improvement (*F* = 18.061, *p* < 0.001). *Post-hoc* comparisons indicated that the improvement in the medium-frequency group was greater than that both the low and high-frequency groups (both *p* < 0.001). To more precisely evaluate the independent effect of FES frequency, a one-way analysis of covariance (ANCOVA) was conducted with post-treatment walking speed as the dependent variable, and pre-treatment walking speed, age, disease duration, and stroke type as covariates. Results showed that, after adjusting for these baseline factors, the main effect of frequency group remained significant (*F* = 16.990, *p* < 0.001). The overall model fit was good (adjusted *R^2^* = 0.886). Further parameter estimates revealed that, compared to the high-frequency group (reference), the medium-frequency group had a significantly higher walking speed (unstandardized coefficient *B* = 0.102, *p* < 0.001), while there was no significant difference between the low and high-frequency groups (*B* = 0.010, *p* = 0.604). This series of data clearly indicates the superior efficacy of medium-frequency FES in improving walking speed in patients with post-stroke FD, an advantage independent of patients’ initial walking capacity, age, disease duration, and stroke type.

**Table 2:** Comparison of 10MWS among the three groups.

Group	Pre-treatment	Post-treatment	Improvement	*p* (Within)	*p* (vs. Medium)
Low-frequency	0.44 ± 0.21	0.60 ± 0.23	0.154 ± 0.063	< 0.001	< 0.001
Medium-frequency	0.42 ± 0.20	0.68 ± 0.24	0.246 ± 0.095	< 0.001	—
High-frequency	0.41 ± 0.20	0.58 ± 0.22	0.145 ± 0.050	< 0.001	< 0.001

Data are presented as mean ± standard deviation. 10MWS, 10-meter maximum walking speed. *p* (Within): from paired t-tests (pre *vs.* post-treatment). One-way ANOVA on improvement scores showed a significant between-group difference (*F* = 18.061, *p* < 0.001). *p* (*vs.* Medium): from post-hoc pairwise comparisons (Least Significant Difference method) against the medium-frequency group. An ANCOVA adjusting for baseline covariates confirmed the between-group effect (*F* = 16.990, *p* < 0.001). No significant difference was found between low and high-frequency groups in improvement (*p* = 0.629) or in the ANCOVA model (*p* = 0.604).

#### 3.2.2 Secondary efficacy outcomes

##### 3.2.2.1 Lower limb motor function: FMA-LE scores

Regarding the FMA-LE scores, which reflect lower limb motor control and coordination, all three groups showed improvement post-treatment compared to pre-treatment (paired *t*-test, all *p* < 0.001). Importantly, the differences in improvement values among groups were statistically significant (*F* = 105.005, *p* < 0.001). Post-hoc pairwise comparisons (LSD) revealed a conclusion highly consistent with the primary outcome: the improvement in the medium-frequency group (8.60 ± 1.99 points) surpassed that of both the low-frequency (3.23 ± 1.36 points) and high-frequency groups (3.53 ± 1.41 points) (both *p* < 0.001), while again no significant difference was found between the low and high-frequency groups (*p* = 0.473). This result suggests that medium-frequency FES may be more efficacious not only in improving functional walking speed but also in facilitating greater overall improvement in lower limb motor impairment, as reflected by the composite FMA-LE score. Details are shown in Table 3.

**Table 3:** Comparison of FMA-LE scores among the three groups.

Group	Pre-treatment	Post-treatment	Improvement	*p* (Within)	*p* (*vs.* Medium)
Low-frequency	12.93 ± 5.21	16.17 ± 5.46	3.23 ± 1.36	< 0.001	< 0.001
Medium-frequency	15.90 ± 4.94	24.50 ± 5.67	8.60 ± 1.99	< 0.001	—
High-frequency	15.53 ± 5.83	19.07 ± 5.80	3.53 ± 1.41	< 0.001	< 0.001

Data are presented as mean ± standard deviation. FMA-LE, Fugl-Meyer Assessment for Lower Extremity. *p* (Within): from paired t-tests (pre *vs.* post-treatment). One-way ANOVA on improvement scores showed a significant between-group difference (*F* = 105.005, *p* < 0.001). *p* (*vs.* Medium): from post-hoc pairwise comparisons (Least Significant Difference method) against the medium-frequency group. No significant difference was found between low and high-frequency groups (*p* = 0.473).

##### 3.2.2.2 Functional walking capacity

For the FAC, an ordinal categorical variable, non-parametric tests showed that the FAC levels post-treatment were significantly higher than pre-treatment levels in all three groups (Wilcoxon signed-rank test, all *p* < 0.001). However, the overall comparison of post-treatment level distribution across the three groups did not reach statistical significance (Kruskal-Wallis H test, *χ^2^* = 5.167, *p* = 0.076). Given this non-significant omnibus test, any subsequent pairwise comparisons should be interpreted with caution as exploratory analyses. Exploratory *post-hoc* pairwise comparisons (Mann–Whitney *U* test) suggested that the functional level in the medium-frequency group might be higher than in the low-frequency group (*p* = 0.043), and showed a non-significant trend towards being higher than the high-frequency group (*p* = 0.060). No difference was suggested between the low and high-frequency groups (*p* = 0.789). Consistent with this directional pattern, the highest mean rank post-treatment was observed in the medium-frequency group (54.07), compared to the low-frequency (40.48) and high-frequency (41.95) groups. These exploratory findings, while indicative of a potential pattern favoring medium-frequency stimulation, require confirmation in larger studies. Results are detailed in Table 4.

**Table 4:** Comparison of FAC among the three groups.

Comparison	Post-treatment mean ranks (group 1 *vs.* group 2)	Mann–Whitney *U* Test *p* value
Medium-frequency *vs.* low-frequency	54.07/40.48	0.043
Medium-frequency *vs.* high-frequency	54.07/41.95	0.060
Low-frequency *vs.* high-frequency	40.48/41.95	0.789

FAC, Functional Ambulation Category. The overall Kruskal-Wallis H test was not statistically significant (*χ*^2^ = 5.167, *df* = 2, *p* = 0.076). Therefore, the pairwise comparisons presented above are exploratory and should be interpreted with caution.

##### 3.2.2.3 Ankle dorsiflexor strength

The results for ankle dorsiflexor strength are summarized in Table 5. Strength, assessed via manual muscle testing (MRC scale), improved significantly from baseline in all three groups (Wilcoxon signed-rank test, all *p* < 0.001). The magnitude of improvement (post-pre difference) differed significantly among the groups (Kruskal-Wallis *H* = 74.771, *p* < 0.001). *Post-hoc* pairwise comparisons with Bonferroni correction revealed that the improvement in the medium-frequency group was significantly greater than that in both the low-frequency group (*p* < 0.001) and the high-frequency group (*p* < 0.001). There was no significant difference in improvement between the low and high-frequency groups (*p* = 0.161). This pattern-superior efficacy of medium-frequency stimulation-is fully consistent with the findings for walking speed (10MWS) and lower limb motor function (FMA-LE). Despite these significant improvements, the post-treatment mean strength across all groups remained within the “Fair” (Grade 3–4) range, indicating that functional gait gains may precede the full recovery of maximal isolated strength. Importantly, the medium-frequency group demonstrated a significantly greater gain in strength compared to the other groups, reinforcing its superior therapeutic profile.

**Table 5:** Comparison of ankle dorsiflexor strength among the three groups.

Group	Pre-treatment	Post-treatment	Improvement	*p* (Within)	*p* (*vs.* Medium)↑
Low-frequency	1.93 ± 1.23	2.90 ± 1.19	0.97 ± 0.18	< 0.001	< 0.001
Medium-frequency	2.00 ± 0.98	3.93 ± 0.87	1.93 ± 0.25	< 0.001	—
High-frequency	1.87 ± 1.33	2.90 ± 1.35	1.03 ± 0.18	< 0.001	< 0.001

Data are presented as mean ± standard deviation. Strength was assessed using the Medical Research Council (MRC) scale. *p* (Within): from Wilcoxon signed-rank tests (pre *vs.* post-treatment). Kruskal-Wallis H test on improvement scores showed a significant between-group difference (*H* = 74.771, *p* < 0.001). ↑P (*vs.* Medium): from *post-hoc* pairwise comparisons (Mann–Whitney *U* test with Bonferroni correction, significance level *p* < 0.017) against the medium-frequency group. No significant difference was found between low and high-frequency groups (*p* = 0.161).

### 3.3 Safety and tolerability analysis

All 90 patients underwent complete safety assessment. Regarding treatment adherence, all enrolled patients completed the full prescribed treatment course, with no early discontinuations, indicating good clinical acceptability for all three FES frequency protocols. As shown in Table 6, a total of 3 mild adverse events were recorded during the entire treatment period, resulting in an overall incidence of 3.3%. Specifically, no adverse events were reported in the low-frequency group; 1 case (3.3%) was reported in the medium-frequency group, presenting as mild local rash; and 2 cases (6.7%) were reported in the high-frequency group, both mild local rashes. All adverse events resolved quickly after adjusting electrode placement or briefly pausing stimulation, and none led to treatment discontinuation. Chi-square test (interpreted considering Fisher’s exact test logic as 50.0% of cells had expected count < 5) indicated no statistically significant difference in adverse event incidence among the three groups (*χ^2^* = 2.069, *p* = 0.355). The combined 100% treatment completion rate and the very low incidence of mild adverse events demonstrate that FES at low, medium, and high frequencies within the parameters used in this study exhibited excellent and comparable clinical safety and tolerability.

**Table 6:** Comparison of adverse event occurrence among the three groups.

Group	No adverse event	Adverse event present	Total
Low-frequency	30 (100.0)	0 (0.0)	30
Medium-frequency	29 (96.7)	1 (3.3)	30
High-frequency	28 (93.3)	2 (6.7)	30
Total	87 (96.7)	3 (3.3)	90

Data are *n* (%). Between-group comparison: *χ*^2^ = 2.069, *p* = 0.355. Interpretation considers Fisher’s exact test due to small expected counts.

### 3.4 Subgroup and sensitivity analyses

To systematically verify the robustness and generalizability of the main findings, sensitivity analyses and subgroup analyses based on stroke type were conducted. Sensitivity analysis using ANCOVA, after controlling for the influence of baseline pre-treatment FMA-LE scores (covariate *p* = 0.686), found that the differences in improvement values among frequency groups remained highly significant (*F* = 100.634, *p* < 0.001). This confirms that the superior improvement in motor function with medium-frequency FES is independent of patients’ initial functional status, supporting the robustness of the core conclusion. Further subgroup analysis results (Table 7) showed that regarding the primary outcome (10MWS improvement), differences among frequency groups were highly significant in the ischemic stroke subgroup (*F* = 14.827, *p* < 0.001), with the medium-frequency group showing significantly greater improvement than both the low and high-frequency groups (both *p* < 0.001). In the hemorrhagic stroke subgroup, the overall difference for the primary outcome (10MWS improvement) was of borderline statistical significance (*F* = 3.349, *p* = 0.057). Therefore, interpretations of the subsequent exploratory *post-hoc* comparisons within this subgroup should be made cautiously. Exploratory *post-hoc* comparisons suggested that the improvement in the medium-frequency group was greater than that in the high-frequency group (*p* = 0.019) and showed a trend towards being better than the low-frequency group (*p* = 0.100). Regarding the secondary outcome (FMA-LE score improvement), the medium-frequency group consistently demonstrated significantly superior improvement in both ischemic and hemorrhagic stroke subgroups (both *p* < 0.001). While the evidence for the primary outcome in the hemorrhagic subgroup was less robust, this cross-outcome pattern provides preliminary, though not conclusive, support for the potential general applicability of the medium-frequency FES efficacy advantage.

**Table 7:** Comparison of FES frequency efficacy in different stroke type subgroups.

Outcome measures	Outcome and subgroup	Statistical test	*P*	Medium *vs.* low	Medium *vs.* high	Low *vs.* high
10MWS improvement	Ischemic stroke	*F* = 14.827	< 0.001	< 0.001	< 0.001	0.984
	Hemorrhagic stroke	*F* = 3.349	0.057	0.100	0.019	0.317
FMA-LE score improvement	Ischemic stroke	*F* = 86.058	< 0.001	< 0.001	< 0.001	0.339
	Hemorrhagic stroke	*F* = 16.867	< 0.001	< 0.001	< 0.001	0.857

FES, functional electrical stimulation; 10MWS, 10-meter maximum walking speed; FMA-LE, Fugl-Meyer Assessment for Lower Extremity; ANOVA, analysis of variance; LSD, least significant difference. Within each stroke subtype, inter-group comparisons of improvement scores were performed using one-way ANOVA. *Post-hoc* pairwise comparisons were performed using the least significant difference (LSD) method.

## 4 Discussion

Stroke is a cerebrovascular disease with complex etiology, characterized primarily by high mortality and disability rates (19, 20). This disease often leads to varying degrees of motor dysfunction in patients, severely impacting their quality of life (21). Consequently, exploring and establishing active and effective rehabilitation interventions post-stroke has become a critical issue in clinical rehabilitation practice, crucial for improving patients’ functional prognosis (22, 23). Among various rehabilitation techniques, electrical stimulation therapy has garnered widespread attention due to its advantages of simplicity and non-invasiveness. Studies indicate that this therapy holds multiple potentials, including reducing muscle atrophy, enhancing muscle strength, increasing joint range of motion, alleviating edema, promoting tissue repair, and relieving pain (24), offering a beneficial approach for post-stroke motor recovery.

This study, through a retrospective cohort analysis, systematically investigated the differential efficacy of FES at different stimulation frequencies when used as an add-on to conventional rehabilitation in patients with post-stroke FD. The core findings consistently indicated that in this combined therapy context, medium-frequency (31–40 Hz) FES was associated with superior outcomes compared to both low and high-frequency stimulation in improving patients’ walking speed and lower limb motor control. A consistent directional trend favoring medium-frequency stimulation was also observed for functional walking capacity (FAC), although the overall inter-group comparison for this ordinal measure did not reach statistical significance. It should be noted that the improvements observed reflect the combined effect of FES and conventional rehabilitation; the study design compares the relative efficacy of different FES frequencies within this additive framework. It is important to emphasize that this study was designed to compare functional efficacy and safety, not to elucidate specific neuromodulatory mechanisms. The efficacy advantage of medium-frequency stimulation was generally present across patients with different stroke types and was not accompanied by an increase in safety risks. Walking speed, which serves as an objective and reliable indicator for assessing functional recovery post-stroke (25, 26), showed significant improvement in the medium-frequency group, providing key evidence for the core conclusion of this study. This finding contributes to the ongoing investigation into optimal FES parameters, suggesting that within the broad effective range of 20–50 Hz commonly used in clinical practice (11), a more refined medium-frequency band may yield superior gait outcomes. In seeking to interpret these findings, we acknowledge that our study design does not allow for definitive causal or mechanistic conclusions. However, the observed pattern of results invites hypothesis generation. One plausible explanation is that the medium-frequency range might represent a therapeutic window that optimally reconciles the need for effective muscle contraction with the constraints of fatigue and patient comfort. It is possible that within the range of frequencies capable of producing functionally fused tetanic contractions (typically >20–25 Hz) (27), the 31–40 Hz band may optimize the force-fatigue trade-off during repetitive, functional use such as walking, compared to either lower or higher extremes within the therapeutic range. This could translate into more consistent foot clearance over a training session. Conversely, while higher frequencies might theoretically produce stronger contractions (as suggested by studies focusing on strength gains (14), they could also be associated with a faster onset of neuromuscular fatigue or reduced tolerability, potentially attenuating their sustained functional benefits over a treatment session. This notion aligns with the differential effects of frequency observed in prior work, where higher frequencies improved strength but lower frequencies were associated with endurance outcomes (14). These remain speculations as we did not directly measure fatigue indices or central neural activity. The superior gains in the composite FMA-LE scores with medium-frequency stimulation could be consistent with better-preserved exercise dose and quality during therapy, potentially facilitating broader improvements in coordinated lower limb motor function. However, as the FMA-LE assesses hip, knee, and ankle movements collectively, this improvement cannot be attributed specifically to the ankle dorsiflexors alone and may reflect enhanced overall limb coordination. This interpretation requires direct verification in future studies with more granular movement analysis.

It is noteworthy that high-frequency FES did not demonstrate superior efficacy compared to low-frequency stimulation. This observation did not align with our pre-study expectation that higher frequencies might yield stronger effects. A post-hoc consideration is that the practical benefits of higher frequencies in a therapeutic context could be offset by factors such as accelerated fatigue or lower patient tolerability, limiting the sustainable dose of effective stimulation during training. This interpretation, while logical, is speculative and underscores the need for research that couples functional outcomes with physiological measures of fatigue and adaptation. Furthermore, this study observed that the efficacy advantage of medium-frequency FES remained stable across both ischemic and hemorrhagic stroke subgroups. This cross-population generalizability enhances the external validity of the study’s conclusion, suggesting this parameter may be suitable for a broad population of patients with post-stroke FD. Regarding safety, all three groups exhibited good tolerability, with adverse events being mild and rare, showing no intergroup differences. This confirms that, within reasonable parameter ranges, pursuing better efficacy did not come at the cost of safety, alleviating safety concerns for the clinical promotion of medium-frequency FES.

The strengths of this study lie in its clear clinical question, rigorous statistical analysis, and multi-level functional assessment indicators. However, as a retrospective study, it also has certain limitations. First and foremost, the non-randomized, retrospective design and the lack of a control group receiving conventional rehabilitation alone limit the internal validity and the ability to establish causality. The allocation to frequency groups was based on clinical practice, and although baseline characteristics were comparable (Table 1), residual confounding cannot be ruled out. The observed improvements reflect the combined effect of FES and background therapy. Second, this study primarily focused on short-term efficacy, lacking follow-up data on the maintenance of long-term effects. Third, the retrospective and clinical-functional design does not permit direct investigation of the physiological or neural mechanisms underlying the differential response to stimulation frequencies. Our discussion of potential reasons is therefore hypothetical. Fourth, the sample size was not uniformly distributed between ischemic and hemorrhagic stroke subtypes, limiting the statistical power of the subgroup analyses, particularly for the hemorrhagic subgroup. Fifth, our functional assessment was limited to clinical outcome measures. Future studies would benefit from including instrumented gait analysis (e.g., kinematics, spatiotemporal parameters) and measures of functional mobility (e.g., Timed Up and Go test) to provide a more comprehensive understanding. Notwithstanding these limitations, this study provides comparative data from real-world clinical practice. Future prospective, large-sample randomized controlled trials that incorporate long-term follow-up, a non-FES control arm, and more objective electrophysiological and biomechanical measures are warranted to both validate our comparative efficacy observations and definitively explore the underlying mechanisms.

## 5 Conclusion

This study demonstrates that among the adjustable parameters of FES for treating post-stroke FD, stimulation frequency is a crucial modifiable factor. Within the commonly used 20–50 Hz range, the findings of this retrospective cohort study suggest that medium-frequency stimulation (31–40 Hz) was associated with the optimal balance between efficacy and safety in our patient population. Based on this evidence, clinical rehabilitation practitioners may consider prioritizing medium-frequency parameters as a potential core choice when formulating FES treatment plans, which could potentially benefit a broad range of patients. This provisional recommendation, derived from comparative effectiveness data, should be viewed alongside the study’s limitations and calls for confirmation through prospective, randomized controlled trials.

## Data Availability

The original contributions presented in the study are included in the article/supplementary material, further inquiries can be directed to the corresponding author.
